# Brief Sensory Training Narrows the Temporal Binding Window and Enhances Long-Term Multimodal Speech Perception

**DOI:** 10.3389/fpsyg.2019.02489

**Published:** 2019-11-05

**Authors:** Michael Zerr, Christina Freihorst, Helene Schütz, Christopher Sinke, Astrid Müller, Stefan Bleich, Thomas F. Münte, Gregor R. Szycik

**Affiliations:** ^1^Department of Psychosomatic Medicine and Psychotherapy, Hannover Medical School, Hanover, Germany; ^2^Department of Psychiatry, Social Psychiatry and Psychotherapy, Hannover Medical School, Hanover, Germany; ^3^Department of Neurology, University of Lübeck, Lübeck, Germany; ^4^Institute of Psychology II, University of Lübeck, Lübeck, Germany

**Keywords:** multisensory integration, speech perception, word recognition, simultaneity judgment, temporal binding, double flash illusion

## Abstract

Our ability to integrate multiple sensory-based representations of our surrounding supplies us with a more holistic view of our world. There are many complex algorithms our nervous system uses to construct a coherent perception. An indicator to solve this ‘binding problem’ are the temporal characteristics with the specificity that environmental information has different propagation speeds (e.g., sound and electromagnetic waves) and sensory processing time and thus the temporal relationship of a stimulus pair derived from the same event must be flexibly adjusted by our brain. This tolerance can be conceptualized in the form of the cross-modal temporal binding window (TBW). Several studies showed the plasticity of the TBW and its importance concerning audio-visual illusions, synesthesia, as well as psychiatric disturbances. Using three audio-visual paradigms, we investigated the importance of length (short vs. long) as well as modality (uni- vs. multimodal) of a perceptual training aiming at reducing the TBW in a healthy population. We also investigated the influence of the TBW on speech intelligibility, where participants had to integrate auditory and visual speech information from a videotaped speaker. We showed that simple sensory trainings can change the TBW and are capable of optimizing speech perception at a very naturalistic level. While the training-length had no different effect on the malleability of the TBW, the multisensory trainings induced a significantly stronger narrowing of the TBW than their unisensory counterparts. Furthermore, a narrowing of the TBW was associated with a better performance in speech perception, meaning that participants showed a greater capacity for integrating informations from different sensory modalities in situations with one modality impaired. All effects persisted at least seven days. Our findings show the significance of multisensory temporal processing regarding ecologically valid measures and have important clinical implications for interventions that may be used to alleviate debilitating conditions (e.g., autism, schizophrenia), in which multisensory temporal function is shown to be impaired.

## Introduction

As Sumby and Pollack (1954) showed more than half a century ago, especially in situations with low signal-to-noise ratios, we utilize visual factors, such as the speakers’ lips and facial movements, to maximize our speech intelligibility. This use of concurrent sensory information from different modalities is plausible on the level of perception and agrees with our everyday experience. On single-cell level Meredith and Stein (1985) were able to show that response from some neurons to stimuli from one specific sensory modality can be influenced by inputs from other modalities. This subpopulation of nerve cells is found in different brain regions that are involved in different functions, but they share substantial similarities (Stein and Wallace, 1996). These *multisensory* neurons have the ability to integrate information about multiple representations of our surrounding and thus supply us with a more holistic picture of what we call ‘reality’. To this regard, it is not arbitrary which stimuli our nervous system will bind together to be different representations of the same ‘object’. There are many different mechanisms our nervous system can use to determine which and in what manner stimuli are processed and integrated to a coherent perception of our world. One indicator for example is the spatial location of the stimuli, meaning that two stimuli are more likely to be attributed to the same source of origin, the more spatially proximate they are (Meredith and Stein, 1986). Analogously, the temporal characteristics are of important value with the specificity that environmental information has different propagation speeds (e.g., sound and electromagnetic waves) and sensory processing time and thus the temporal relationship of a stimulus pair derived from the same event must be adjusted by our multisensory system (Meredith et al., 1987). This tolerance for temporal co-occurrence of stimuli from different sensory modalities can be conceptualized in the form of the *multimodal temporal binding window (TBW)*. The average TBWs for typically developing adults range from 160 ms for simple audio-visual stimuli (flash/beep) to 250 ms for more complex stimuli like speech (Wallace and Stevenson, 2014).

A widened TBW was demonstrated to occur in autism (Mongillo et al., 2008; Russo et al., 2010; Donohue et al., 2012; de Boer-Schellekens et al., 2013; Woynaroski et al., 2013; Zmigrod et al., 2013; Stevenson et al., 2014a,b,c), developmental dyslexia (Bastien-Toniazzo et al., 2010; for a critical discussion about the specific disease mechanism see Hairston et al., 2005; Francisco et al., 2017) and schizophrenia (De Gelder et al., 2003, 2005; Foucher et al., 2007; Ross et al., 2007b; De Jong et al., 2009; Pearl et al., 2009; Hass et al., 2017; Zvyagintsev et al., 2017). Zmigrod and Zmigrod (2016) showed additionally that a narrower TBW was associated with a better performance in verbal and non-verbal problem-solving tasks in a healthy population.

Studies have shown the short- and longer-term malleability of the TBW. The former is mostly referred to as *recalibration* and can be induced by an exposure to asynchronous stimuli for a certain time, which results in a ‘lag adaptation’ in the sensory processing system (e.g., Fujisaki et al., 2004; Vroomen et al., 2004; Navarra et al., 2005; Hanson et al., 2008). To their own surprise Powers et al. (2009) induced a narrowing of the multimodal TBW. The changes persisted over a period of seven days. The authors used a perceptual learning paradigm in which participants were given feedback during a two-alternative forced choice audiovisual simultaneity judgement task (SJT) and could exclude the possibility of changes in cognitive biases as the underlying mechanism. Another study showed similar results using an unisensory training (Stevenson et al., 2013). In both studies, most of the effect was seen after one training session, raising the question whether there is the need for multiple iterations of the procedure. Using another paradigm as a criterion Fujisaki et al. (2004) demonstrated that the recalibration of the TBW altered the temporal tuning in an audio-visual illusion. Furthermore (2016) investigated the effects of a multisensory training on a sound-induced flash illusion (SIFI) and found a correlation between the degree of TBW narrowing and increases in sensitivity (d-prime), but no improvements in response bias. On the other hand (2012) as well as (2014) found a reduction of susceptibility to an audio-visual illusion with improvements in multisensory temporal processes. Surig et al. (2018) varied the task difference of a two-alternative forced-choice SJT (either at each participant’s individual threshold or randomly chosen) and discovered faster improvements in the ‘adaptive’ condition regarding the processing speed of auditory inputs as well as the size of the ventriloquist effect. De Niear et al. (2016) altered the task difficulty of a SJT and observed that enhancements in temporal acuity could be optimized by employing audio-visual stimuli for which it is difficult to judge temporal synchrony. In another study, De Niear et al. (2018) showed that perceptual training was capable of enhancing temporal acuity for simple stimuli (‘flashes’ and ‘beeps’) as well as for more complex speech stimuli (the phoneme ‘ba’). However, they failed to observe a generalization across levels of stimulus complexity.

The investigations carried out so far showed the plasticity of the TBW and its importance concerning audio-visual illusions, problem-solving tasks, dyslexia, as well as severe psychiatric disturbances with an early onset, like autism and schizophrenia (for an overview see Wallace and Stevenson, 2014). Thus, using specific training paradigms to influence the width of TBW could be interesting to reduce multisensory deficits of the previous mentioned populations.

In the current study we address different issues related to both the malleability of the TBW and the generalization effects of the trained sensory modality and also of the potential effects on speech perception. Based on the aforementioned investigations, we hypothesized that a long- and short-term training, regardless of their modality, should have no different effects on the narrowing of the TBW. An exploratory hypothesis was formulated concerning the role of the modality (uni- vs. multimodal training). Last, we assumed that a narrowing of the TBW should have a positive effect on speech intelligibility - more precisely in situations with low signal-to-noise ratios, where you would expect people to benefit from seeing the speakers’ lips and facial movements.

## Materials and Methods

All procedures had been approved by the local Ethics Committee of the Hannover Medical School and have been performed in accordance with the ethical standards laid down in the Declaration of Helsinki. The participants gave written informed consent and participated for a small monetary compensation.

### Participants

A total of 40 subjects (age *M* = 22.60 years, *SD* = 3.50, range = 20–37; females = 23) participated in the study. Five additional control subjects (age *M* = 30.00 years, *SD* = 6.29, range = 21–38; females = 2) were involved in a short experiment to control for possible effects resulting from repeated presentation of speech stimuli. Participants were mostly undergraduate and graduate students of biology and biochemistry as well as psychologists. Only subjects with normal or corrected to normal vision and normal hearing were included. No participant had a history of neurological or psychiatric diseases. In all cases German was the native language.

We randomized the subjects in four groups equal in size (*n* = 10). To control for possible differences regarding intelligence (especially the crystallized intelligence), a multiple-choice vocabulary intelligence test ‘Mehrfachwahl-Wortschatz-Intelligenztest – MWT-B’ (Lehrl, 2005) was used. The MWT-B consists of 37 items arranged by difficulty. Every item consists of one word as defined by the German dictionary as well as four fictitious words. Participants have to indicate the ‘real’ word by underlining it. The test took about 5 min to complete.

### Stimuli and Design

**FIGURE 1:**
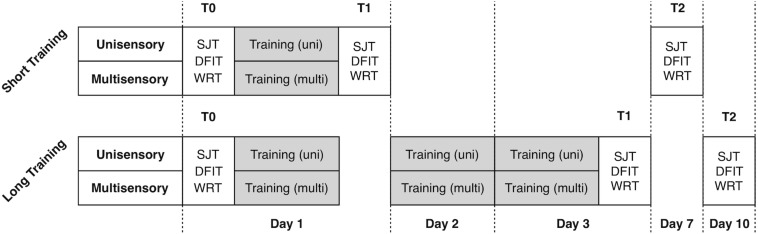
Timeline of the experimental procedure. 40 participants were randomly assigned to the four experimental training conditions.

**FIGURE 2:**
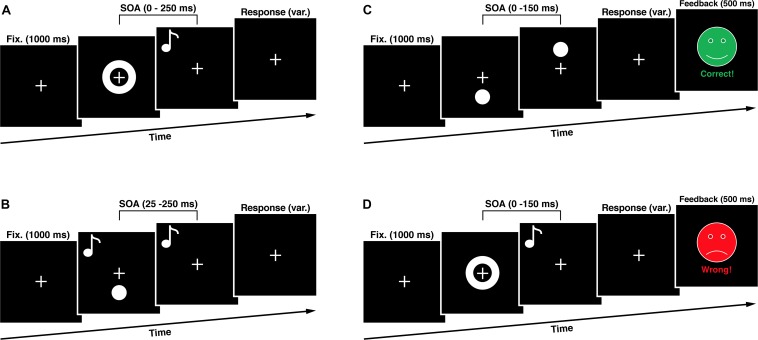
Experimental Sequences. **(A)** In the simultaneity judgment task (SJT) participants had to judge whether an audio-visual stimulus pair was presented synchronous or asynchronous. **(B)** In the double flash illusion task (DFIT) participants indicated perceiving one or two visual flashes. **(C)** The unisensory training consisted of a visual simultaneity judgment task with feedback. **(D)** The multisensory training consisted of an audio-visual simultaneity judgment task with feedback. SOA, stimulus onset asynchrony.

The study included analysis of four experimental groups in 2 × 2 design with factors training-length (short vs. long) and training-modality (unisensory vs. multisensory). For the training we used only visual (unimodal) and audiovisual (multisensory) modifications of the SJT. The training length varied between only one training unit and three units on three consecutive days. To assess the training effects, we used three well-established audio-visual paradigms, namely the SJT, the Double Flash Illusion Task (DFIT) and the Word Recognition Task (WRT). We collected the data before the first training (T0, first day), after the training (T1, first respective third day) and seven days after (T2). Between each measurement/training, participants had the chance to rest for maximum 5 min. During experiments, the investigator observed the behavior of the subject via video stream, beyond that there was no interaction between subjects and the examiner. Participants were advised to make a decision as fast as possible and otherwise to guess the right decision. The paradigms as well as the timeline of the experimental procedure are depicted in Figures 1, 2 and described in more detail beyond.

#### SJT

To assess the audio-visual temporal processing, we used a task in which participants judge whether a visual and an auditory stimulus were presented synchronous or asynchronous by pressing appropriate button on a response device. Visual stimuli consist of a white ring (6 deg. of visual angle) on a black background presented for one refresh cycle (8.3 ms) in the center of the visual field. The auditory stimulus was an 1850 HZ tone presented for 8 ms at stimulus onset asynchronies (SOAs) in relation to the visual stimulus onset ranging from 0 to 250 ms at 25 ms intervals constituting one synchronous and ten asynchronous SOA conditions. We used only visual-leading conditions. A total of 165 trials in pseudorandom order made up the task resulting in 15 trials per SOA condition. Between the stimuli presentations a white fixation cross (2 deg. of visual angle) on a black background was presented in the middle of the visual field for 1000 ms. The whole paradigm duration was 189 s plus the variable time for the subjects’ response. Subjects indicated perceiving the stimuli as synchronous or asynchronous by pressing either the button of the left or the right response device.

#### DFIT

The cross-modal double flash illusion, also called the SIFI, occurs, when two short auditory stimuli (inducers) are presented in quick succession accompanied by a single visual flash (target) and these auditory stimuli are perceptually grouped by being attributed to the same source of origin (Roseboom et al., 2013). In this case, the illusionary perception of an additional visual flash (fission illusion) manifests itself (Shams et al., 2000, 2002). During the whole experiment a white fixation cross (2 deg. of visual angle) on a black background was presented in the middle of the visual field. To induce the illusion, a white flash (4 deg. of visual angle) on a black background was presented in the peripheral visual field (4 deg. beyond the center of the fixation cross) accompanied by two sound beeps (1850 Hz, 8 ms in duration) with SOAs ranging from 25 to 250 ms at 25 ms intervals. We decided to present the flashes in the peripheral visual field because is known that this results in the strongest induction of the fission illusion (Shams et al., 2002). A total of 170 trials in pseudorandom order made up the task resulting in 15 trials per SOA condition with an additional 20 trials consisting of two control conditions with presentation of ‘one flash, one beep’ as well as ‘two flashes, no beep’. Subjects indicated perceiving one or two flashes by pressing either the button of the left or the right response device. The whole paradigm duration was 216 s plus the variable time for the subjects’ response.

#### WRT

We used the same WRT as Sinke et al. (2014). This task contains german high frequency disyllabic lemmas derived from the CELEX-Database (Baayen et al., 1995) with a Mannheim frequency 1.000.000 (MannMln). The MannMln frequency indicates the down scaled occurrence of the selected word per one million words taken from the Mannheim 6.0-million-word corpus. The videotaped stimuli were spoken by a male native speaker of german with linguistic experience. Each stimulus had a duration of 2-s showing the frontal view of the speaker’s face. For the auditory-alone condition, the video stream was replaced with a frozen image of the speaker’s face. The audio-visual condition comprised stimuli with synchronous auditory and visual speech information. In addition, the audio stream of both conditions was mixed with white noise of either 0 or 12 dB resulting in a total of four conditions (0 dB audio, 12 dB audio, 0 dB audio-visual and 12 dB audio-visual). Twenty words were used for each of the four WRT-conditions resulting in a total of 80 stimuli. All stimuli were presented in a pseudorandom order. The experimental procedure was designed according to Ross et al. (2007a). After the presentation of each stimulus the subjects were asked to report which word they understood. If a word was not clearly understood, they were instructed to guess the word. Otherwise they should report ‘I did not understand anything’. The answer was recorded by the experimenter. Any answer different from the presented stimulus was counted as false, meaning only whole-word recognitions was counted as correct. When the answer was given, the experimenter triggered the next trial which began with a fixation cross 1 s of duration followed by the next stimulus.

#### Multisensory Training

The multisensory training task was designed according to Powers et al. (2009) and differed in one key aspect from the SJT used in our study, as it contained a feedback. The subject was presented with either the phrase “Correct!” paired with a happy green face, or “Incorrect” paired with a sad red face corresponding to the correctness of their choice. These faces (8 deg. of visual angle) were presented in the center of the visual field for 500 ms after the response of the subject. For the training only SOAs between 0 and 150 ms, in 25 ms intervals, were used. In addition, the veridical simultaneous condition (SOA 0 ms) had a 6:1 ratio to any of the other six non-simultaneous conditions creating an equal likelihood of simultaneous/non-simultaneous conditions and thus minimizing concerns about response bias. There were 120 trials presented pseudorandomly in the training phase (60 times SOA 0 ms condition and 10 times each other SOA condition). We only used visual-leading conditions for two reasons. On the one hand, we tried to keep the cognitive load of our subjects as low as possible to ensure enough concentration for the whole experiment. Therefore we decided to use a small amount of trials to shorten the experimental tasks duration. On the other hand, there is growing evidence that visual- and auditory-leading stimulus compositions are based on different multisensory sampling mechanisms. The auditory-leading condition presents itself as non-malleable and the effects of the visual-leading condition seem to be non-transferable to it (Cecere et al., 2016), which was also demonstrated by Powers et al. (2009) and Stevenson et al. (2013). A plausible explanation for this asymmetry is the fact that, because of the substantial higher transmission speeds of electromagnetic waves, auditory-leading conditions never occur in nature and thus never had to be flexibly specified by the nervous system.

#### Unisensory Training

Our unisensory training was designed with the same timing structure as the multisensory training in this study but contained only visual stimuli (visual flashes), which had to be judged regarding their synchronicity. Visual stimuli (4 deg. of visual angle) were presented 4 deg. of visual angel underneath and above the fixation mark. There were 120 trials presented pseudorandomly in the training phase (60 times SOA 0 ms condition and 10 times each other SOA condition).

Considering the findings of Powers et al. (2009) as well as Stevenson et al. (2013), who demonstrated the necessity of feedback for inducing long-lasting changes in the TBW, we decided to use feedback for our subjects regarding the synchronicity of the stimuli within the SJT training units. Both, the unisensory and multisensory training had duration of approximately four to 5 min depending on the response times of the participants.

All stimuli were presented binaural via loudspeakers placed beside of a high refresh rate monitor (Sony Multiscan G520, 120 Hz) placed in a quiet room approximately 60 cm in front of the subjects. All auditory stimuli were presented at individual subjective level of good audibility. Presentation software (Neurobehavioral Systems, Inc., Albany, CA, United States, version 14.9) was used to control all experiments and collect data.

### Data Analysis

The effects of the four training conditions on the SJT, the DFIT and the WRT were examined using several univariate repeated measures analyses of variance (ANOVAs) followed by *post hoc* t-tests with correction for multiple comparisons (Bonferroni). To investigate possible repetition-effects of the WRT, we used the non-parametric Friedman-Test. If parametric tests were used, they met the assumptions. In cases, were the assumption of sphericity was not met (i.e., significant Mauchly Test), depending on the magnitude of ε, we used either the Greenhouse-Geisser (ε < 0.75) or the Huynh-Feldt (ε > 0.75) correction according to (Girden, 1992). The SJT was used to estimate a TBW. This window was defined to represent the *x*-value of the intersection between the equation *y* = 0.75 (75% frequency of simultaneity judgment) and a sigmoidal function (Eq. I) generated by Matlab R2017b (MathWorks, Natick, MA, United States) to fit the empirical data (see also Powers et al., 2009; Hillock-Dunn and Wallace, 2012).

(1) $$s\,i\,g\,\left(x\right)=\frac{1}{1+e^{\frac{x+\alpha }{\beta }}}$$

## Results

### Baseline

To rule out the possibility of effects driven by mechanisms (e.g., floor or ceiling effects or insufficient randomization) other than implied by our hypotheses, we tested whether the randomization procedure created comparable groups regarding all dependent measures. Several one-way ANOVAs were computed showing no significant differences at the first point of measurement for all of the 11 SJT-, 10 DFIT- und 4 WRT-Conditions (for full statistics, we refer to our Supplementary Material). On average, there were no significant differences regarding age (*F* (_3_, _36_) = 0.375, *p* = 0.771, η^2^_p_ = 0.03), gender (χ^2^ = 4.289, *p* = 0.232, η^2^_p_ = 0.04) as well as performance in the MWT-B (*F* (_3_, _36_) = 1.845, *p* = 0.156, η^2^_p_ = 0.13) across the four experimental groups. There was no attrition bias and no non-compliant participant behavior ensuring treatment integrity throughout the whole experiment. Furthermore, there was no data missing.

### Simultaneity Judgment Task

Using the above-mentioned equation, we derived TBW’s for each participant although we did not use them for the assessment of a potential training effect due to a strongly varying quality of goodness of fit (adjusted R-square ranging from 0.1 to 0.9). Instead, we used SOA’s to track increases in performance in the SJT and report TBW’s online at group-level, where they showed to have a high goodness of fit (adjusted R-square ranging from 0.92 to 0.98).

To measure the effect of the training on the performance in the SJT, we conducted an univariate repeated measures analysis of variance with point of measurement and SOA as within-subjects factors and training-modality and training-length as between-subjects factors.

As expected, SOA had a significant main effect on SJT-performance (*F* (_1.939_, _69_._804_) = 115.625, *p* < 0.001, η^2^_p_ = 0.76) meaning that subjects had higher accuracies in simultaneity judgments as SOA increased. Also point of measurement had a significant main effect (*F* (_1.763_, _63.456_) = 52.684, *p* < 0.001, η^2^_p_ = 0.59), with accuracies improving after training. Furthermore, training-modality revealed a main effect (*F* (_1_, _36_) = 10.731, *p* = 0.002, η^2^_p_ = 0.23), with higher accuracies in simultaneity judgments in the multisensory trainings. Training-length, however, remained insignificant (*F* (_1_, _36_) = 1.333, *p* = 0.256, η^2^_p_ = 0.04), thus all following calculations were collapsed across the factor training-length. Looking at the first-order-interaction effects, point of measurement and training-modality showed a significant effect (*F* (_1.763_, _63.456_) = 14.666, *p* < 0.001, η^2^_p_ = 0.29) with higher accuracies in the multisensory trainings after the training. Also there was a significant effect between SOA and training-modality (*F* (_1.939_, _69.804_) = 4.250, p = 0.019, η^2^_p_ = 0.11) with higher accuracies in the multisensory trainings with increasing SOA’s. Also there was a significant effect between point of measurement and SOA (*F* (_11.028_, _369.992_) = 7.135, *p* < 0.001, η^2^_p_ = 0.17) with higher accuracies after the training with increasing SOA’s.

**FIGURE 3:**
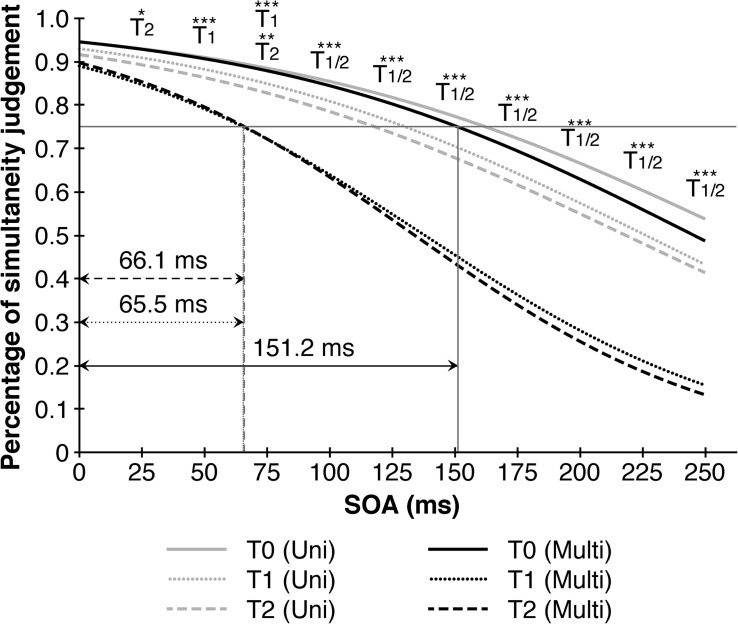
Multisensory trainings narrows audio-visual temporal binding window in comparison to unisensory training. Depicted are significant differences in multisensory trainings between different points of measurement in reference to the 1th point of measurement (T0) resulting in a substantial decrease in the temporal binding window from 151.2 ms (T0) to 65.5 ms (T1) resp. 66.1 ms (T2). Black lines represent multisensory trainings, gray lines represent unisensory trainings. SOA = Stimulus onset asynchrony. ^∗^*p* < 0.05. ^∗∗^*p* < 0.01. ^∗∗∗^*p* < 0.001.

Taking point of measurement, SOA and training-modality into consideration, a significant second-order interaction (*F* (_11.028_, _369.992_) = 2.263, *p* = 0.002, η^2^_p_ = 0.07) indicated performance benefits in simultaneity judgments after the multisensory trainings at higher SOA’s. A closer examination revealed that the multisensory training contributed consistently to a better performance between pre-training (T0) and post-training (T1) as well as T0 and follow up (T2) across all SOAs > 25 ms, but not between T1 and T2 denoting a stable effect over the course of seven days. The improvements contributed to a decrease of the TBW’s in the multisensory group from 151.2 ms (T0) to 65.5 ms (T1), respectively 66.1 ms (T2) compared to only a slight reduction from 162.3 ms (T0) to 130.2 ms (T1) and 118.0 ms (T2) in the unisensory group (Figure 3).

On the other hand, the unisensory training revealed only one significant effect between T1 and T2 at SOA = 25 ms. To analyze the possible generalization effect from the unisensory to multisensory modality, we examined the data from the trainings itself. First, we compared the accuracy of judgments between the three training sessions regarding training-modality. Therefore, only the both long trainings with more than one training session were included (*N* = 20). For simplicity, we collapsed all SOA-conditions creating an indicator for the overall-performance. A univariate repeated measures analysis of variance with point of measurement as a within-subjects factor and training-modality as a between-subjects factor revealed a main effect of point of measurement (*F* (_1.166_, _20.995_) = 13.408, *p* < 0.001, η^2^_p_ = 0.43) and training-modality (*F* (_1_, _18_) = 10.988, *p* = 0.004, η^2^_p_ = 0.38) as well as a significant interaction effect of both factors (*F* (_1.166_, _20.995_) = 5.550, *p* = 0.024, η^2^_p_ = 0.24). A closer look showed that between-training-improvements took place between the first and second (*T* (_19_) = 3.167, *p* = 0.005, *d* = 71) as well as between the first and third (*T* (_19_) = 3.644, *p* = 0.002, *d* = 0.81), but not between the second and third training session (*T* (_19_) = −0.130, *p* = 0.898, *d* = 0.03). Furthermore, these improvements were only noticeable after the multisensory training. We cross-checked these results by comparing the overall-performance between the first vs. the second half of the training at T0. Therefore, all subjects could be included. A univariate repeated measures analysis of variance with the first and second half of the training data at T0 as a within-subjects factor as well as training-modality and training-length as between-subjects factors revealed more accurate judgments in the second half of the training (*F* (_1_, _36_) = 72.552, *p* < 0.001, η^2^_p_ = 0.67) as well as a higher performance in the multisensory trainings (*F* (_1_, _36_) = 9.951, *p* = 0.003, η^2^_p_ = 0.22). Training-length (*F* (_1_, _36_) = 0.142, *p* = 0.708, η^2^_p_ < 0.01) as well as the interaction of training-modality and training-length failed to reach significance (*F* (_1_, _36_) = 0.946, *p* = 0.337, η^2^_p_ = 0.03). While the interaction of the first and second half of the training and training-length (*F* (_1_, _36_) = 0.130, *p* = 0.720, η^2^_p_ < 0.01) as well as the second-order interaction of all three factors remained insignificant (*F* (_1_, _36_) = 0.321, *p* = 0.575, η^2^_p_ = 0.01), the interaction of the first and second half of the training and training-modality showed a significant effect (*F* (_1_, _36_) = 44.259, *p* < 0.001, η^2^_p_ = 0.55), meaning, there were within-session-improvements only in the multisensory training.

### Double Flash Illusion Task

**FIGURE 4:**
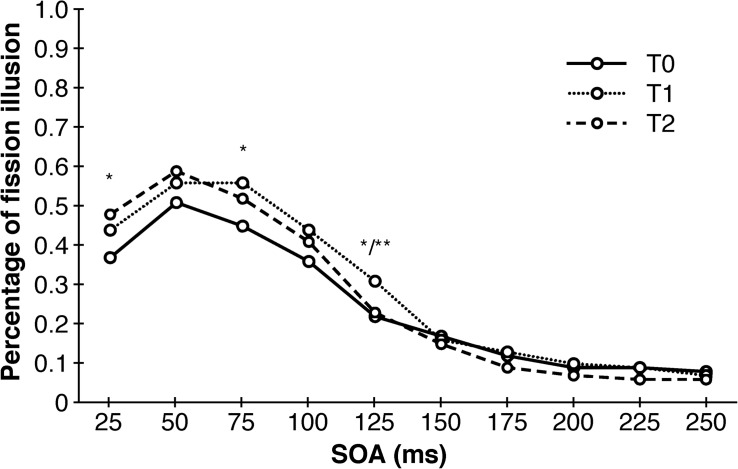
Sensory trainings have no consistent effects on the occurrence of the double-flash illusion. Depicted are significant differences between different points of measurements collapsed across training-modality and training-length. SOA, stimulus onset asynchrony. ^∗^*p* < 0.05. ^∗∗^*p* < 0.01.

An univariate repeated measures analysis of variance with point of measurement and SOA as within-subjects factors and training-modality and training-length as between-subjects factors revealed a main effect of SOA (*F* (_1.713_, _66.666_) = 51.394, *p* < 0.001, η^2^_p_ = 0.59), an first-order interaction effect of SOA and point of measurement (*F* (_6.929_, _249.126_) = 3.551, *p* = 0.001, η^2^_p_ = 0.09) as well as a second-order interaction of SOA, training-length and training-modality (*F* (_1.713_, _61.666_) = 3.691, *p* = 0.037, η^2^_p_ = 0.09). The main effect of SOA points to a less frequent occurrence of the double-flash illusion as SOA increases, which was expected. The significant interaction of SOA and point of measurement revealed a total of four significant *post hoc* tests, but without any consistent pattern whatsoever: there was a significant decrease of illusions between T0 and T2 at SOA = 25 (*T* (_39_) = 2.828, *p* = 0.025, *d* = 0.45), between T0 and T1 at SOA = 75 ms (*T* (_39_) = 2.566, *p* = 0.029, *d* = 0.41), between T0 and T1 at SOA = 125 ms (*T* (_39_) = 2.802, *p* = 0.022, *d* = 0.44) as well as a significant increase in illusion between T1 and T2 also at SOA = 125 ms (*T* (_39_) = −3.189, *p* = 0.009, *d* = 0.50). Similarly, this was the case for the three-way interaction with only one significant *post hoc* test (*T* (_18_) = −1.524, *p* = 0.046, *d* = 0.72). The results of the DFIT unraveled by training-modality are depicted in Figure 4.

### Word Recognition Task

To measure the effect of the training on the performance in the WRT, we conducted an univariate repeated measures analysis of variance with point of measurement and WRT-condition as within-subjects factors and training-modality and training-length as between-subjects factors.

**FIGURE 5:**
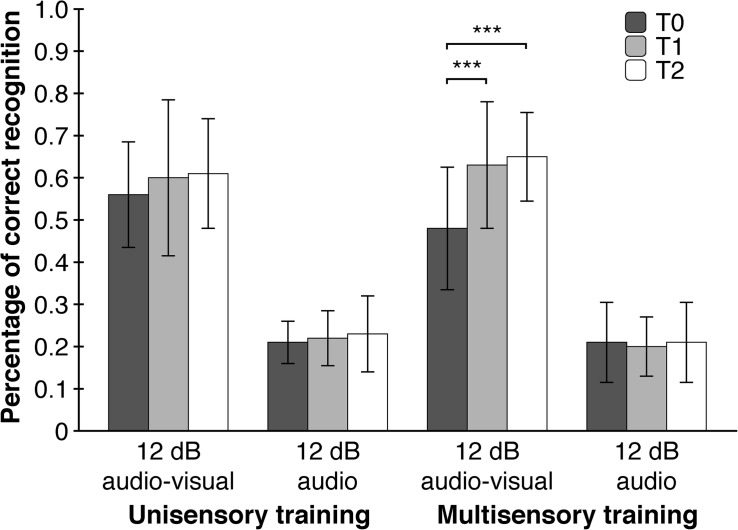
Multisensory trainings contribute to an increased performance in word recognition in situations with a low signal-to-noise ratio (12 dB audio-visual). Depicted are two of the four WRT conditions of interest. Error Bars indicate SDs. ^∗∗∗^*p* < 0.001.

As hypothesized, point of measurement had a significant main effect on WRT-performance (*F* (_1.897_, _68.295_) = 12.453, *p* < 0.001, η^2^_p_ = 0.26) meaning that subjects had higher accuracies in word recognition after the training. As expected, subjects differed in WRT-performance between the four WRT-conditions (*F* (_1.328_, _47.814_) = 459.216, *p* < 0.001, η^2^_p_ = 0.93). A significant interaction effect of point of measurement and WRT-Condition (*F* (_3.617_, _130.215_) = 7.118, *p* = 0.002, η^2^_p_ = 0.17) showed that the effect of the training was apparent only in the ‘12 db audio-visual’ – condition with significant improvements between T0 and T1 (*T* (_39_) = −3.968, *p* < 0.001, *d* = 0.63) as well as T0 and T2 (*T* (_39_) = −4.773, *p* < 0.001, *d* = 0.75), but not between T1 and T2 (*T* (_39_) = −0.834, *p* = 0.409, *d* = 0.13). A second-order interaction effect between point of measurement, WRT-condition and training-modality (*F* (_3.617_, _130.215_) = 3.560, *p* = 0.034, η^2^_p_ = 0.09), implicating a moderating role of training-modality, showed significant improvements in word recognition between T0 and T1 (*T* (_19_) = −4.199, *p* < 0.001, *d* = 0.94) as well as T0 and T2 (*T* (_19_) = −5.403, *p* < 0.001, *d* = 1.21), but not between T1 and T2 (*T* (_19_) = −0.873, *p* = 0.394, *d* = 0.20). These improvements took place only in the ‘12 db audio-visual’ – condition and only after the multisensory trainings. Furthermore, we tested whether each of the four WRT-conditions differed with respect to training-length and training-modality. We assumed a higher WRT-performance only in the ‘12 dB - audio-visual’ - condition due to the just mentioned significant training effect and a lack of baseline-differences at T0. To our surprise, the four WRT-conditions did not show differences regarding training-length and training-modality at T1 and T2. A closer examination of the WRT-data showed, though not significant, a substantial lower baseline-level (η^2^_p_ = 0.11) in the ‘12 dB audio-visual’ – condition in the multisensory group compared to the unisensory group at T0 (Figure 5).

To rule out the possibility that differences in performance are based solely on the repetition of the WRT, five additional participants accomplished the WRT three times without any training. A Friedman-Test for dependent measures revealed no significant differences in the ‘12 dB audio-visual condition’ across the three points of measurement (χ^2^ (_2_) = 0.471, *p* = 0.790, *W* = 0.05). Additionally, we compared the first and second half of the ‘12 dB audio-visual’ data at T0. Should there be a repetition effect, which manifests itself in a higher WRT-performance at post-training, than this should also be the case when comparing the first and second half within the pre-training data. An univariate repeated measures analysis of variance with the first and second half of the ‘12 dB audio-visual’ WRT-data from pre-training as a within-subjects factor and training-modality and training-length as between-subjects factors failed to reach significance for the main effect of WRT (*F* (_1_, _36_) = 0.000, *p* > 0.999, η^2^_p_ = 0.000) as well as all interaction effects (see Supplementary  Material).

## Discussion

In the current study, we demonstrated that a short multisensory training can change the cross-modal TBW and is capable of enhancing speech perception at a very naturalistic level over the course of at least 7 days. Based on previous research results we hypothesized, trainings longer in duration should have no additional effect on TBW. Indeed, both long trainings showed no performance advantages over the short trainings, which is represented by insignificant main and interaction effects with the factor length. This finding stands in line with Powers et al. (2009), who observed significant effects after a single day of training with no incremental performance benefit with repetition. While our multisensory trainings induced a strong narrowing of the TBW, the unisensory trainings failed to do so. This contradicts the results of Stevenson et al. (2013) although we had a higher statistical power due to a higher number of subjects completing the training (*n* = 14 vs. *n* = 20 in our study). One major difference between both studies concerns the use of different paradigms: In our study, participants had to judge the synchronicity of two stimuli. Stevenson et al. used a temporal order judgment task (TOJT), where participants were instructed to indicate which of the two presented visual stimuli appeared first. Like Schneider and Bavelier (2003) pointed out, the TOJT and SJT are prone to different response biases. In our training, subjects may anticipate stimuli to be more likely synchronous just because of the instruction to judge the synchronicity. In the TOJT, participants may believe that stimuli never appear simultaneous because the temporal order has to be judged. Another difference concerns the selection of SOAs: The range of SOAs in the study of Stevenson et al. was smaller (−37,5 to 37,5 ms compared to 0 to 250 ms in our study), which might have led to a stronger training effect, because most of our SOA’s could have been out of range for contributing to a training effect in a visual SJT. Another difference concerns the number of trials: In our study, a total number 120 trials were presented compared to 780 trials in the study of Stevenson et al. (2013), which might have led to a much weaker training effect in our study. Despite the same number of trials, this obviously was not the case for the multisensory training, which might point to a facilitation effect due to bimodal information processing.

The main finding of our study was the generalization effect of the multisensory training on speech perception. We assumed that a narrowing of the TBW should have a positive effect on speech intelligibility in situations with a low signal-to-noise ratio, where informations from an additional modality enhances comprehensibility and therefore an optimized multimodal processing is advantageous. Indeed, a narrowing of the TBW after multisensory trainings was associated with a 33.9% increase in WRT-performance in the ‘12 dB audio-visual’ – condition compared to an 8.1% increase in WRT-performance after unisensory trainings (collapsed across the second and third point of measurement). Interestingly, WRT-performance in the ‘12 dB audio-visual’ – condition did not differ between uni- and multi-sensory trainings at T1 and T2. A closer examination of the WRT-data showed, though not significant, a substantial lower baseline-level (η^2^_p_ = 0.11) in the ‘12 dB audio-visual’ – condition in the multisensory group compared to the unisensory group at T0. This non-significant lower baseline combined with an also non-significant difference in WRT-performance in the ‘12 dB audio-visual’ – condition at T1 and T2 between the different training-modalities could ‘enable’ a statistically significant training-effect to emerge. The lower baseline limits the interpretability of the training effect, although the difference in correct word recognition between the unisensory (55.5% correct recognitions) and multisensory group (47,5% correct recognitions) at T0 seems to be too small for a floor effect of such a size to arise. The fact that the significant training effect in the multisensory group is associated with an improvement in SJT supports its validity.

Despite the narrowing of the TBW, we failed to observe an effect on the DFIT, which is in line with the investigation of Powers et al. (2016) but contradicts the results of Stevenson et al. (2012) as well as Setti et al. (2014). An explanation for the effect of a narrowed TBW on speech perception and its absence on the DFIT concerns possible differences in signal processing mechanism underlying the WRT and DFIT. In the WRT, the presentation of visual stimuli (lip movements) influences the processing of auditory information (speech). In the DFIT (especially in the fission illusion) sound stimuli impacts visual perception.

Our findings have imported clinical implications regarding severe psychiatric conditions like autism and schizophrenia, where a widened TBW was demonstrated to occur (for example Stevenson et al., 2014c; Hass et al., 2017). Because even healthy subjects benefit from our training regarding speech intelligibility, one can assume that subjects with a chronically widened TBW would do so even more. Patients with schizophrenia show a variety of deficits in the processing of multisensory information, such as a smaller facilitation effect of lip reading on auditory speech information (De Gelder et al., 2003; Ross et al., 2007b). This ‘perceptual incoherence’ may give rise to incoherent self-experiences including depersonalization, ambivalence, diminished sense of agency and ‘loosening of associations’ between thoughts (Postmes et al., 2014). Our short multisensory training could be used to address this perceptual incoherence in people with schizophrenia by reducing the TBW. On the other hand, subjects with autism spectrum disorder (ASD) also show audio-visual integration deficits (Feldman et al., 2018). Worse performance in this population is more pronounced in younger subjects and is correlated with autism symptom severity. But deficits in audio-visual speech perception seem to disappear in early adolescence (Foxe et al., 2015). Thus, multisensory integration problems may be directly related to disturbed or prolonged maturation of the sensory system in ASD (Brandwein et al., 2015; Beker et al., 2018). In these cases, multisensory training as used in our study and applied to young subjects with ASD may influence in positive manner performance of audio-visual integration and contribute to reduction of symptom severity in this population. Another open question concerns the generalizability of our findings to younger as well as older populations, where multisensory deficits are occurring.

Our study had several important restrictions, limiting its conclusiveness. The SOA’s used in our unisensory training could have been out of range for a substantial training effect to arise, thus underestimating the impact of the unisensory training. Taking the results of De Niear et al. (2016) into account, our training procedures could have been optimized by employing an adaptive algorithm that automatically selects SOA’s based on every participant’s unique threshold. This approach could have led to smaller SOA’s in our unisensory training and therefore ultimately to a significant effect. Another important limitation relates to the SIFI. We only assessed the fission illusion neglecting the possible effect of the TBW on the fusion illusion, which should be considered more differentiated. Another important issue is related to a not optimal goodness of fit deriving the TBW’s on an individual level from our SJT. Our findings would have a stronger explanatory power, if we had observed a significant correlation between the degree of TBW-narrowing and improvement in the WRT.

Future research should try to replicate the generalization effect of simple audio-visual trainings on speech perception with individually adopted SOA’s, or at least using significant reduced SOA distances. This appears to be relevant because both variables could be related in a non-linear fashion. This notion is supported by an investigation of Sinke et al. (2014), where the authors observed a reduced speech perception in subjects with synesthesia, which are known to have a narrower TBW then the general population. This would imply the existence of an ‘optimal’ TBW with deviations in both ways leading to detrimental effects regarding speech intelligibility, constituting an inverted U-shape.

## Data Availability Statement

The datasets generated for this study are available on request to the corresponding author.

## Ethics Statement

The studies involving human participants were reviewed and approved by Ethics Committee of the Hannover Medical School. The patients/participants provided their written informed consent to participate in this study.

## Author Contributions

MZ performed the measurements, participated in the statistical analysis and interpretation of the data, and drafted the manuscript. CF and HS participated in the coordination of the study and performed the measurement. CS participated in the coordination of the study, study design, and statistical analysis. AM and SB participated in the interpretation of the data. TM participated in the study design and interpretation of the data. GS conceived the study, participated in the study design and coordination, and statistical analysis and interpretation of the data, and drafted the manuscript. All authors read and approved the final manuscript.

## Conflict of Interest

The authors declare that the research was conducted in the absence of any commercial or financial relationships that could be construed as a potential conflict of interest.
