# Supplementary material for: Brief Sensory Training Narrows the Temporal Binding Window and Enhances Long-Term Multimodal Speech Perception
Source: Front Psychol. 2019 Nov 5;10:2489. doi: 10.3389/fpsyg.2019.02489 (PMC6848860; doi:10.3389/fpsyg.2019.02489)
Supplement: Supplementary file 1 [file Table_1.DOCX]

|  | df | Mean of squares | F | *p* | 𝜂^2^_p_ |
| --- | --- | --- | --- | --- | --- |
| **SJT** |  |  |  |  |  |
| SOA = 0ms | 3 | 1.292 | .573 | .636 | .046 |
| SOA = 25ms | 3 | .667 | .210 | .889 | .017 |
| SOA = 50ms | 3 | .567 | .264 | .851 | .021 |
| SOA = 75ms | 3 | 3.158 | .737 | .537 | .058 |
| SOA = 100ms | 3 | .292 | .099 | .960 | .008 |
| SOA = 125ms | 3 | 6.300 | .833 | .484 | .065 |
| SOA = 150ms | 3 | 9.667 | 1.016 | .397 | .078 |
| SOA = 175ms | 3 | 8.825 | .622 | .605 | .049 |
| SOA = 200ms | 3 | 28.167 | 1.777 | .169 | .129 |
| SOA = 225ms | 3 | 17.758 | 1.366 | .269 | .102 |
| SOA = 250ms | 3 | 26.467 | 1.257 | .304 | .095 |
| **DFIT** |  |  |  |  |  |
| SOA = 25ms | 3 | 27.300 | 1.392 | .261 | .104 |
| SOA = 50ms | 3 | 31.892 | 1.429 | .250 | .106 |
| SOA = 75ms | 3 | 24.292 | 1.108 | .358 | .085 |
| SOA = 100ms | 3 | 14.567 | .666 | .578 | .053 |
| SOA = 125ms | 3 | 5.233 | .383 | .766 | .031 |
| SOA = 150ms | 3 | 1.267 | .107 | .955 | .009 |
| SOA = 175ms | 3 | 4.633 | .592 | .624 | .047 |
| SOA = 200ms | 3 | 7.200 | 1.080 | .370 | .083 |
| SOA = 225ms | 3 | 8.100 | 1.309 | .287 | .098 |
| SOA = 250ms | 3 | 7.067 | 1.005 | .402 | .077 |
| **WRT** |  |  |  |  |  |
| 0 dB audio-visual | 3 | .067 | .387 | .763 | .031 |
| 0 dB visual | 3 | .300 | .514 | .675 | .041 |
| 12 dB audio-visual | 3 | 11.533 | 1.505 | .230 | .114 |
| 12 dB visual | 3 | 2.425 | .958 | .423 | .038 |

Table 1: Differences regarding all conditions of the three dependent variables between the four experimental groups at T0.

Table 2: univariate repeated measures analysis of variance with point of measurement and SOA (SJT) as within-subjects factors and training-modality and training-length as between-subjects factors.

|  | df | Mean of squares | F | *p* | 𝜂^2^_p_ |
| --- | --- | --- | --- | --- | --- |
| **point of measurement** | 1.763 | 844.052 | 52.684 | <.001* | .594 |
| **point of measurement** X **training-length** | 1.763 | .548 | .034 | .953 | .001 |
| **point of measurement** X **training-modality** | 1.763 | 234.965 | 14.666 | <.001* | .289 |
| **point of measurement** X **training-length** X **training-modality** | 1.763 | 2.342 | .146 | .839 | .004 |
| **SOA** | 1.939 | 5259.193 | 115.615 | <.001* | .763 |
| **SOA** X **training-length** | 1.939 | 9.886 | .217 | .799 | .006 |
| **SOA** X **training-modality** | 1.939 | 193.313 | 4.250 | .019* | .106 |
| **SOA** X **training-length** X **training-modality** | 1.939 | 48.410 | 1.064 | .349 | .029 |
| **point of measurement** X **SOA** | 11.028 | 37.363 | 7.135 | <.001* | .165 |
| **point of measurement** X **SOA** X **training-length** | 11.028 | 2.704 | .516 | .893 | .014 |
| **point of measurement** X **SOA** X **training-modality** | 11.028 | 14.048 | 2.683 | .002* | .069 |
| **point of measurement** X **SOA** X **training-length** X **training-modality** | 11.028 | 7.677 | 1.466 | .142 | .039 |
| **training-length** | 1 | 13.516 | 1.333 | .256 | .036 |
| **training-modality** | 1 | 108.819 | 10.731 | .002* | .230 |
| **training-length** X  **training-modality** | 1 | 31.752 | 3.131 | .085 | .080 |

Table 3: Differences in simultaneity judgements regarding training-modality, SOA (SJT) and point of measurement.

|  | Mean difference | *p* | Cohens *d* |  | Mean difference | *p* | Cohens *d* |
| --- | --- | --- | --- | --- | --- | --- | --- |
| **Unisensory Training** |  |  |  | **Multisensory Training** |  |  |  |
| SOA = 0ms |  |  |  | SOA = 0ms |  |  |  |
| T0-T1 | .30 | >.999 | .18 | T0-T1 | .15 | >.999 | .06 |
| T0-T2 | .25 | >.999 | .15 | T0-T2 | .20 | >.999 | .08 |
| T1-T2 | -.05 | >.999 | .03 | T1-T2 | .05 | >.999 | .04 |
| SOA = 25ms |  |  |  | SOA = 25ms |  |  |  |
| T0-T1 | -.25 | >.999 | .15 | T0-T1 | .65 | .540 | .27 |
| T0-T2 | .75 | .387 | .42 | T0-T2 | 1.35 | .025* | .55 |
| T1-T2 | 1.00 | .027* | .57 | T1-T2 | .70 | .183 | .49 |
| SOA = 50ms |  |  |  | SOA = 50ms |  |  |  |
| T0-T1 | .60 | .627 | .36 | T0-T1 | 2.00 | <.001* | .81 |
| T0-T2 | .75 | .499 | .62 | T0-T2 | .85 | .355 | .28 |
| T1-T2 | .15 | >.999 | .09 | T1-T2 | -1.15 | .090 | .42 |
| SOA = 75ms |  |  |  | SOA = 75ms |  |  |  |
| T0-T1 | .80 | .511 | .36 | T0-T1 | 2.30 | <.001* | .79 |
| T0-T2 | 1.00 | .297 | .58 | T0-T2 | 2.35 | <.001* | .68 |
| T1-T2 | .20 | >.999 | .10 | T1-T2 | .50 | >.999 | .02 |
| SOA = 100ms |  |  |  | SOA = 100ms |  |  |  |
| T0-T1 | 1.10 | .263 | .38 | T0-T1 | 3.20 | <.001* | 1.17 |
| T0-T2 | 1.40 | .240 | .59 | T0-T2 | 3.55 | <.001* | .82 |
| T1-T2 | .30 | >.999 | .12 | T1-T2 | .35 | >.999 | .12 |
| SOA = 125ms |  |  |  | SOA = 125ms |  |  |  |
| T0-T1 | .50 | >.999 | .25 | T0-T1 | 4.20 | <.001* | 1.06 |
| T0-T2 | 1.15 | .415 | .39 | T0-T2 | 4.90 | <.001* | 1.34 |
| T1-T2 | .65 | .947 | .23 | T1-T2 | .70 | .841 | .26 |
| SOA = 150ms |  |  |  | SOA = 150ms |  |  |  |
| T0-T1 | 1.25 | .372 | .44 | T0-T1 | 5.15 | <.001* | 1.29 |
| T0-T2 | 1.40 | .239 | .47 | T0-T2 | 4.95 | <.001* | 1.31 |
| T1-T2 | .15 | >.999 | .06 | T1-T2 | -.20 | >.999 | .07 |
| SOA = 175ms |  |  |  | SOA = 175ms |  |  |  |
| T0-T1 | 1.55 | .071 | .63 | T0-T1 | 5.00 | <.001* | 1.48 |
| T0-T2 | 1.20 | .243 | .38 | T0-T2 | 4.35 | <.001* | 1.63 |
| T1-T2 | -.35 | >.999 | .11 | T1-T2 | -.65 | .992 | .24 |
| SOA = 200ms |  |  |  | SOA = 200ms |  |  |  |
| T0-T1 | .65 | .855 | .27 | T0-T1 | 5.00 | <.001* | 1.70 |
| T0-T2 | 1.65 | .111 | .49 | T0-T2 | 5.85 | <.001* | 1.69 |
| T1-T2 | 1.10 | .248 | .43 | T1-T2 | .85 | .413 | .33 |
| SOA = 225ms |  |  |  | SOA = 225ms |  |  |  |
| T0-T1 | 1.25 | .122 | .56 | T0-T1 | 4.60 | <.001* | 1.57 |
| T0-T2 | 1.75 | .074 | .54 | T0-T2 | 5.25 | <.001* | 1.60 |
| T1-T2 | .50 | >.999 | .17 | T1-T2 | .65 | .748 | .34 |
| SOA = 250ms |  |  |  | SOA = 250ms |  |  |  |
| T0-T1 | 2.00 | .085 | .62 | T0-T1 | 4.45 | <.001* | .95 |
| T0-T2 | 2.15 | .052 | .60 | T0-T2 | 5.10 | <.001* | 1.25 |
| T1-T2 | .15 | >.999 | .06 | T1-T2 | .65 | .647 | .35 |

Table 4: univariate repeated measures analysis of variance with point of measurement and SOA (DFIT) as within-subjects factors and training-modality and training-length as between-subjects factors.

|  | df | Mean of squares | F | *p* | 𝜂^2^_p_ |
| --- | --- | --- | --- | --- | --- |
| **point of measurement** | 1.768 | 41.065 | 2.930 | .067 | .075 |
| **point of measurement** X **training-length** | 1.768 | 38.181 | 2.725 | .080 | .070 |
| **point of measurement** X **training-modality** | 1.768 | 15.893 | 1.134 | .232 | .031 |
| **point of measurement** X **training-length** X **training-modality** | 1.768 | 29.590 | 2.112 | .135 | .055 |
| **SOA** | 1.713 | 5130.150 | 51.394 | <.001* | .588 |
| **SOA** X **training-length** | 1.713 | 14.982 | .150 | .829 | .004 |
| **SOA** X **training-modality** | 1.713 | 137.648 | 1.379 | .258 | .037 |
| **SOA** X **training-length** X **training-modality** | 1.713 | 368.471 | 3.691 | .037* | .093 |
| **point of measurement** X **SOA** | 6.920 | 22.885 | 3.551 | .001* | .090 |
| **point of measurement** X **SOA** X **training-length** | 6.920 | 7.958 | 1.235 | .285 | .033 |
| **point of measurement** X **SOA** X **training-modality** | 6.920 | 7.728 | 1.199 | .304 | .032 |
| **point of measurement** X **SOA** X **training-length** X **training-modality** | 6.920 | 7.716 | 1.197 | .305 | .032 |
| **training-length** | 1 | 21.505 | .879 | .355 | .024 |
| **training-modality** | 1 | 3.333 | .136 | .714 | .004 |
| **training-length** X  **training-modality** | 1 | 13.601 | .556 | .461 | .015 |

Table 5: univariate repeated measures analysis of variance with point of measurement and WRT as within-subjects factors and training-modality and training-length as between-subjects factors.

|  | df | Mean of squares | F | *p* | 𝜂^2^_p_ |
| --- | --- | --- | --- | --- | --- |
| **point of measurement** | 1.897 | 21.533 | 12.699 | <.001* | .261 |
| **point of measurement** X **training-length** | 1.897 | 1.906 | 1.124 | .328 | .030 |
| **point of measurement** X **training-modality** | 1.897 | 3.874 | 2.285 | .112 | .060 |
| **point of measurement** X **training-length** X **training-modality** | 1.897 | 1.168 | .689 | .498 | .019 |
| **WRT** | 1.328 | 14952.472 | 1354.132 | <.001* | .974 |
| **WRT** X **training-length** | 1.328 | 3.721 | .337 | .627 | .009 |
| **WRT** X **training-modality** | 1.328 | .408 | .037 | .907 | .001 |
| **WRT** X **training-length** X **training-modality** | 1.328 | 4.423 | .401 | .588 | .011 |
| **point of measurement** X **WRT** | 3.617 | 21.643 | 7.402 | <.001* | .171 |
| **point of measurement** X **WRT** X **training-length** | 3.617 | 1.101 | .377 | .806 | .010 |
| **point of measurement** X **WRT** X **training-modality** | 3.617 | 8.317 | 2.845 | .031* | .073 |
| **point of measurement** X **WRT** X **training-length** X **training-modality** | 3.617 | .484 | .165 | .944 | .005 |
| **training-length** | 1 | .434 | .803 | .376 | .022 |
| **training-modality** | 1 | .156 | .289 | .594 | .008 |
| **training-length** X  **training-modality** | 1 | .434 | .803 | .376 | .022 |

Table 6: Differences regarding all four WRT-conditions between the four experimental groups at T1 and T2.

|  | df | Mean of squares | F | *p* | 𝜂^2^_p_ |
| --- | --- | --- | --- | --- | --- |
| **T1** |  |  |  |  |  |
| **0 db audio-visual** |  |  |  |  |  |
| training-length | 1 | .025 | .059 | .810 | .002 |
| training-modality | 1 | .025 | .059 | .810 | .002 |
| training-length X training-modality | 1 | .025 | .059 | .810 | .002 |
| **0 db visual** |  |  |  |  |  |
| training-length | 1 | .100 | .194 | .663 | .005 |
| training-modality | 1 | .000 | .000 | >.999 | .000 |
| training-length X training-modality | 1 | .400 | .774 | .385 | .021 |
| **12 db audio-visual** |  |  |  |  |  |
| training-length | 1 | 12.100 | 1.048 | .313 | .028 |
| training-modality | 1 | 3.600 | .312 | .580 | .009 |
| training-length X training-modality | 1 | .900 | .078 | .782 | .002 |
| **12 db visual** |  |  |  |  |  |
| training-length | 1 | 2.025 | 1.046 | .313 | .028 |
| training-modality | 1 | 1.225 | .633 | .432 | .017 |
| training-length X training-modality | 1 | .025 | .013 | .910 | .000 |
| **T2** |  |  |  |  |  |
| **0 db audio-visual** |  |  |  |  |  |
| training-length | 1 | .025 | .164 | .688 | .005 |
| training-modality | 1 | .225 | 1.473 | .233 | .039 |
| training-length X training-modality | 1 | .025 | .164 | .688 | .005 |
| **0 db visual** |  |  |  |  |  |
| training-length | 1 | .025 | .093 | .752 | .003 |
| training-modality | 1 | .225 | .835 | .367 | .023 |
| training-length X training-modality | 1 | .025 | .093 | .752 | .003 |
| **12 db audio-visual** |  |  |  |  |  |
| training-length | 1 | .225 | .036 | .851 | .001 |
| training-modality | 1 | 7.225 | 1.154 | .290 | .031 |
| training-length X training-modality | 1 | 1.225 | .196 | .661 | .005 |
| **12 db visual** |  |  |  |  |  |
| training-length | 1 | .625 | .182 | .673 | .005 |
| training-modality | 1 | 1.225 | .356 | .555 | .010 |
| training-length X training-modality | 1 | 3.025 | .879 | .355 | .024 |

Table 7: univariate repeated measures analysis of variance with the first and second half of the 12db-audio-visual data from WRT at T0 as within-subjects factors and training-modality and training-length as between-subjects factors.

|  | df | Mean of squares | F | *p* | 𝜂^2^_p_ |
| --- | --- | --- | --- | --- | --- |
| **WRT (first vs. second half)** | 1 | .000 | .000 | >.999 | .000 |
| **WRT (first vs. second half)** X **training-length** | 1 | .200 | .091 | .764 | .003 |
| **WRT (first vs. second half)** X **training-modality** | 1 | .200 | .091 | .764 | .003 |
| **WRT (first vs. second half)** X **training-length** X **training-modality** | 1 | .800 | .365 | .549 | .010 |
| **training-length** | 1 | .200 | .042 | .838 | .001 |
| **training-modality** | 1 | 3.200 | .676 | .416 | .018 |
| **training-length** X **training-modality** | 1 | 5.000 | 1.056 | .311 | .029 |

Table 8: Stimulus list of the disyllabic words used in the WRT.

| Abend | Besitz | Figur | Kammer | Partner | Umfang |
| --- | --- | --- | --- | --- | --- |
| Ablauf | Besuch | Finger | Karte | Pause | Umsatz |
| Absatz | Betrag | Flugzeug | Kasse | Person | Umstand |
| Abschluss | Betrieb | Folge | Kenntnis | Planung | Urlaub |
| Abschnitt | Beweis | Forschung | Kontakt | Presse | Urteil |
| Absicht | Bezirk | Fortschritt | Konzert | Problem | Verband |
| Abstand | Bilanz | Foto | Kosten | Prozent | Verein |
| Alter | Bildung | Freiheit | Küche | Prozess | Verkauf |
| Anfang | Bischoff | Freundschaft | Lage | Rahmen | Verkehr |
| Anlass | Boden | Garten | Lager | Reform | Verlag |
| Ansicht | Börse | Garten | Leben | Regel | Verlauf |
| Anspruch | Bühne | Gebiet | Lehre | Regen | Verlust |
| Anteil | Bürger | Gedicht | Leistung | Reihe | Vernunft |
| Antrag | Büro | Gefahr | Leiter | Reise | Versuch |
| Antwort | Bursche | Gefühl | Leitung | Richter | Vertrag |
| Anzahl | Chance | Gegend | Leser | Richtung | Vogel |
| April | Dauer | Gerät | Leute | Rolle | Vorbild |
| Arbeit | Dichter | Gericht | Lösung | Roman | Vorgang |
| Armee | Drittel | Geschäft | Mangel | Schaden | Vorjahr |
| Aufbau | Ehe | Gesetz | Mannschaft | Schatten | Vorstand |
| Auftrag | Ehre | Gesicht | Masse | Schreiben | Vorteil |
| Auge | Eindruck | Gespräch | Mauer | Schule | Vortrag |
| August | Einheit | Gestalt | Meister | Schüler | Wagen |
| Ausbau | Einsatz | Gewicht | Meldung | Seite | Wahrheit |
| Auskunft | Einsicht | Gewinn | Menge | Sendung | Ware |
| Ausschuss | Eltern | Glaube | Metall | Sitzung | Wasser |
| Aussicht | Empfang | Größe | Meter | Spannung | Wesen |
| Auto | Ende | Gründung | Mittel | Spieler | Wetter |
| Autor | Entschluss | Gruppe | Modell | Spreche | Wille |
| Bahnhof | Entwurf | Hafen | Moment | Stärke | Willen |
| Basis | Erde | Haltung | Motor | Stimme | Wirkung |
| Bauer | Erfolg | Handel | Musik | Straße | Wohnung |
| Bedarf | Ertrag | Handlung | Nachbar | System | Zeichen |
| Beginn | Fachmann | Haushalt | Name | Tasche | Zeitschrift |
| Begriff | Fahrer | Herrschaft | Nummer | Termin | Zeitung |
| Beifall | Fahrzeug | Hilfe | Nutzen | Titel | Zentrum |
| Beitrag | Farbe | Himmel | Oper | Tonne | Zimmer |
| Bereich | Fehler | Hörer | Ordnung | Tote | Zone |
| Bericht | Fenster | Jugend | Organ | Treffen | Zustand |
| Beruf | Feuer | Kaffee | Partei | Trommel | Zweifel |
